# Adaptive liquid interfaces induce neuronal differentiation of mesenchymal stem cells through lipid raft assembly

**DOI:** 10.1038/s41467-022-30622-y

**Published:** 2022-06-03

**Authors:** Xiaofang Jia, Jingwen Song, Wenyan Lv, Jonathan P. Hill, Jun Nakanishi, Katsuhiko Ariga

**Affiliations:** 1 School of Pharmaceutical Sciences (Shenzhen), Shenzhen Campus of Sun Yat-sen University, Shenzhen, 518107 China; 2 International Center for Materials Nanoarchitectonics (MANA), National Institute for Materials Science (NIMS), 1-1 Namiki, Tsukuba, Ibaraki 305-0044 Japan; 3 Department of Advanced Materials Science, Graduate School of Frontier Sciences, The University of Tokyo, 5-1-5 Kashiwanoha, Kashiwa, Chiba 277-8561 Japan; 4 Research Center for Functional Materials, National Institute for Materials Science, 1-1 Namiki, Tsukuba, Ibaraki 305-0044 Japan

**Keywords:** Biomaterials - cells, Self-assembly, Mesenchymal stem cells, Biomedical engineering

## Abstract

Stem cells and their microenvironment interact cooperatively to dictate their fates. Biomaterials are dynamically remodeled by stem cells, and stem cells sense and translate the changes into cell fate decisions. We have previously reported that adaptive biomaterials composed of fibronectin inserted into protein nanosheets at a liquid interface enhance neuronal differentiation of human mesenchymal stem cells (hMSCs). However, we could not decouple clearly the effect of ligand density from that of fibrillary structure on cellular function and fate. Here we present an adaptive biomaterial based on two-dimensional networks of protein nanofibrils at a liquid–liquid interface. Compared with flat protein nanosheets, this biomaterial enhances neuronal differentiation of hMSCs through a signaling mechanism involving focal adhesion kinase. Lipid raft microdomains in plasma membrane are found to play a central role in which hMSCs rapidly adapt to the dynamic microenvironment at the fluid interface. Our finding has substantial implications for regenerative medicine and tissue engineering.

## Abstract (web summary)

In this work the authors report how human mesenchymal stem cells rapidly adapt to dynamic microenvironment through lipid raft in membrane microdomains that direct neurogenesis.

## Introduction

Biophysical cues of extracellular milieu have a profound effect on the regulation of cell behavior and function^1^. Static properties of substrates, such as their inherent stiffness, nanotopography and geometry, have been widely investigated to explore how biophysical cues drive stem cell lineage specification^2,3^. Besides these well-established parameters, matrix viscoelastic property with stress relaxation has been further proven to be critical in controlling cell fate and activity^4,5^. In native tissues, cells do not passively perceive and respond to signals from their niche. Stem cells and extracellular matrix (ECM) continuously mutually adapt. That is, stem cells remodel the ECM, and the inherent properties of ECM dictate stem cell fate^6^. Currently available dynamic biomaterials rely largely on external stimuli such as light, electrical, and magnetic fields, which trigger the presence or absence of adhesive ligand, or produce two-state switching in their stiffness^7–12^. However, the native extracellular microenvironment, which displays dynamic adaptive behaviors, goes far beyond the stimuli-triggered two-state switching in their properties^13^.

Nature is not limited to solid media and liquid state adaptive systems are ubiquitous^14^. In the human body, surfaces such as those of eyes, lungs and joints, are fused and lined with liquids. Liquids can flow and actively redistribute, allowing the creation of a completely new set of dynamic adaptive systems with potential applications in many realms from reconfigurable ferromagnetic liquid droplets to anti-fouling medical devices^14,15^. Recently, we have developed a bio-inspired adaptive interface formed from a centimeter-sized self-assembled protein nanosheet at a liquid–liquid interface^16^. Adaptive to cell traction force, protein assemblies based on fibronectin undergo an ultrastructure transition from nanosheets to hierarchical fibers as a result of interfacial jamming. These results led to the hypothesis that elongated fibronectin fibers at a liquid–liquid interface facilitate enhanced cytoskeletal tension and neuronal differentiation. However, we cannot clearly decouple the effect of ligand density from that of fibrillary structure on cellular function and stem cell fate, and how it directs hMSCs fate remains unexplored.

**Fig. 1:**
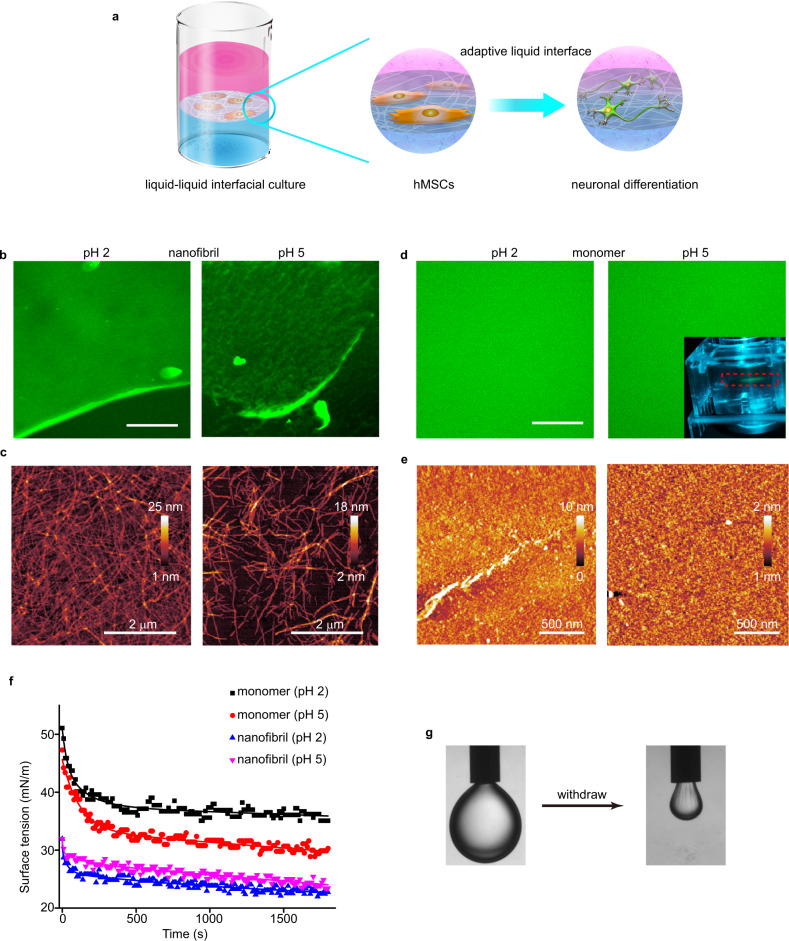
An adaptive biomaterial based on self-assembled protein nanostructures on a liquid–liquid interface. **a** Schematic of neuronal differentiation of hMSCs at the interfacially assembled two-dimensional network of protein nanofibrils. **b** Fluorescence images of ThT-dyed lysozyme nanofibrils assembly at the PFO/water interface at pH 2 and pH 5. Scale bar: 100 μm. **c** Representative AFM images of lysozyme nanofibrils assembled at the PFO/water interface at pH 2 and pH 5. **d** Fluorescence images of FITC-lysozyme assembly at the PFO/water interface at pH 2 and pH 5. Scale bar: 100 μm. Inset shows the optical photograph of protein layer at the PFO/water interface under blue light irradiation. **e** Representative AFM images of protein nanosheets at the PFO/water interface at pH 2 and pH 5. **f** Time evolution of interfacial tension between lysozyme monomer/nanofibril aqueous solution and PFO at pH 5 and pH 2. **g** Images of the interfacial jamming process when PFO phase is withdrawn from the droplet in the bath of lysozyme nanofibrils solution at pH 5.

Protein nanofibrils, which are polymeric β-sheet aggregates of protein on the order of a few micrometers long, are excellent candidates for the mimicking of ECM matrix fibers such as collagen, owing to their biocompatibility for cell attachment, and high mechanical strength^17,18^. Interest in protein nanofibrils is burgeoning from the points of view of tissue engineering and drug encapsulation^19,20^. Herein, we construct an adaptive biomaterial based on two-dimensional networks of protein nanofibrils at a liquid–liquid interface. In parallel, we examine self-assembled protein nanosheets at a liquid–liquid interface. In contrast to flat protein nanosheets, hMSCs show enhanced neuronal differentiation when cultured on two-dimensional networks of protein nanofibrils at a liquid–liquid interface (Fig. 1a). We hypothesize that cells interpret biophysical cues at the liquid–liquid interface involving the lipid raft/focal adhesion kinase (FAK) pathway, which directs the neuronal differentiation of hMSCs.

## Results and discussion

### Preparation of the adaptive liquid interface

To visualize the self-assembly behavior of protein nanofibrils and protein monomers at different pHs at a liquid–liquid interface, we have adopted a fluorescent labeling method. Protein nanofibrils were obtained by thermal incubation of hen egg-white lysozyme at 90 °C^21^. As shown in Supplementary Fig. 1, the resulting nanofibrils have diameters of about 3 nm and lengths in the range 0.5–10 μm. We examined the distribution of the nanofibrils at a liquid–liquid interface by labeling the nanofibrils with thioflavine T (ThT). As shown in Fig. 1b, the in-situ fluorescence microscopy image reveals that protein nanofibrils at pH 2 are closely packed at the liquid interface. However, at pH 5, the nanofibrils are sparsely adsorbed at the interfaces. Atomic force microscopy (AFM) images indicate surface coverages of protein nanofibrils of 76.8% and 31.1% at pH 2 and 5, respectively (Fig. 1c). At pH 5, the adsorption of protein nanofibrils at liquid interfaces takes place at a greater rate due to the decreased charge-charge repulsion between the protein nanofibrils, resulting in a loose and disordered alignment^22^. At pH 2, the slower adsorption rate allows the unperturbed in-plane rotational rearrangement, leading to an ordered and close-packed alignment. In contrast, at the same mass concentration of proteins, native lysozyme monomers formed a centimeter-sized perfect protein nanosheet at a liquid interface, as revealed by the in-situ fluorescence microscopy images of fluorescein isothiocyanate labeled lysozyme (Fig. 1d). The corresponding height profiles derived from AFM images indicate that the thicknesses of protein monolayers are 1.8 ± 0.1 nm and 2.3 ± 0.3 nm at pH 5 and 2, respectively, which agrees well with the lysozyme structural model (4.5 × 3 × 3 nm, an ellipsoidal shape) (Supplementary Fig. 2)^23^. Root mean square (rms) roughness on the sub-nanometer range (0.14 ± 0.01 nm for pH 5, and 0.11 ± 0.02 nm for pH 2) implies that a homogeneous protein monolayer is closely packed at the liquid interface (Fig. 1e). These findings indicate that the interfacial packing of these protein nanofibrils and protein monomers can be manipulated by adjusting the pH.

To gain insight into the molecular mechanism of lysozyme interfacial self-assembly, Fourier transform infrared (FTIR) spectra were collected (Supplementary Fig. 3). At pH 5, adsorption of lysozyme monomers to PFO leads to a 12.8% loss of α-helix and a corresponding 13.6% increase in β-sheet similar to the secondary-structure of protein nanofibrils, indicating an amyloid-like structure of the protein nanosheets (Supplementary Table 1). At pH 2, lysozyme chains are partially unfolded due to interruption of hydrogen bonding under acidic conditions. As expected, lysozyme was adsorbed at PFO with the complete loss of α-helix and partial β-sheet structure, accompanied by a significant increase in random structures. The larger amide I/II ratio at pH 2 consistently shows a greater degree of protein conformational change than that at pH 5^24^. Thus, lysozyme is self-assembled at PFO mainly through β-sheet packing at pH 5 and through interprotein hydrophobic interactions at pH 2^25^.

We further investigated the kinetics of assembly and interfacial jamming of the lysozyme monomers/nanofibrils at the liquid–liquid interface by using pendant drop tensiometry. As shown in Fig. 1f, the equilibrium interfacial tension of protein monomers is lower at pH 5 than at pH 2, implying denser, stiffer protein assemblies at pH 5^26^. At pH 5, a second slower decrease of interfacial tension after an initial rapid decay was observed, indicated a secondary structure rearrangement from α-helix to β-sheet at the interface. For protein nanofibrils, a lower equilibrium interfacial tension at pH 2 indicates denser alignment at the interface, consistent with the AFM and fluorescence images (Fig. 1b, c). As shown in Fig. 1g and Supplementary Fig. 4, visible wrinkles can be observed when the PFO phase is withdrawn from the droplet. Upon decrease of the interfacial area, protein assemblies are compressed, ultimately causing protein assemblies to jam. Proteins are jammed at the interface, leading to elastic behavior, and undergo a liquid-like to solid-like phase transition establishing enhanced mechanical properties^26,27^.

Self-assembly features of the protein nanosheets at a liquid–liquid interface cause it to adapt dynamically to cell traction forces. As shown in Supplementary Fig. 5, obvious ridges were observed along cell peripheries after 1 day of culture. As the cells spread progressively at the interface, actomyosin contractility-driven tension pulls the protein nanosheets centripetally, causing visible ridges. When cell traction force is released, the interfacial area is decreased, and protein assemblies are compressed. Protein assemblies then jam at the interface, going through a fluid-like to solid-like transition leading to adaptively enhanced mechanical properties^28^. The interfacial jamming process arrests morphology variation and stabilizes the liquid–liquid interface with a hierarchical fiber network. Furthermore, longer fibrillary structures are observed in the protein nanosheets obtained at pH 5 than that obtained at pH 2. The larger proportion of β-sheet stacking of protein nanosheets at pH 5 presumably facilitates the morphology transition to longer fibrillary structures relative to the prevalent random stacking observed at pH 2.

### Mechanosensing of hMSCs at liquid interface

**Fig. 2:**
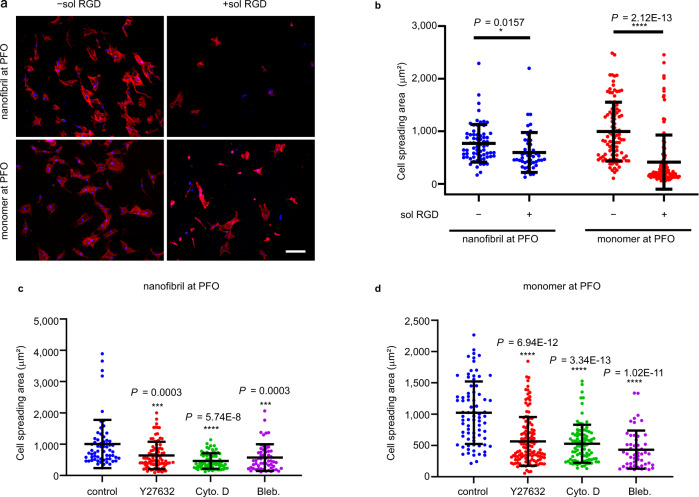
Cell adhesion and spreading at adaptive liquid interface through molecular clutch model. **a** Representative images of hMSCs on the monolayers of lysozyme nanofibril and monomer at the PFO/water interface (pH 2) after 2 h of no treatment, or treatment with soluble RGD (sol RGD, 2 mM). **b** Quantification of cell spreading area. *n* = 67 (−sol RGD, nanofibril at PFO), 45 (+sol RGD, nanofibril at PFO), 100 (−sol RGD, monomer at PFO), and 110 (+sol RGD, monomer at PFO) cells from 2 biologically independent experiments. Lines and error bars indicate the mean ± s.d. *P < 0.05, ****P < 0.0001, one-way ANOVA with Bonferroni *post hoc*. Quantification of cell spreading area of hMSCs on the monolayers of lysozyme nanofibril (**c**) and monomer (**d**) at the PFO/water interface (pH 2) after 2 h of no treatment (control), or treatment with an inhibitor; **c** *n* = 62 (control), 86 (Y27632), 75 (Cyto.D) and 57 (Bleb.) cells, **d** *n* = 78 (control), 125 (Y27632), 91 (Cyto.D) and 50 (Bleb.) cells, from 2 biologically independent experiments. **c**–**d**, Lines and error bars indicate the mean ± s.d. ***P < 0.001, ****P < 0.0001 versus control, one-way ANOVA with Bonferroni *post hoc*.

Next, we sought to investigate cell proliferation and spreading on the interfacially assembled (IA) lysozyme monolayers and lysozyme nanofibrils. Cell viability assays indicated that this set of materials supports cell survival and cell toxicity was not observed (Supplementary Fig. 6). Staining for the incorporation of thymidine analogue EdU indicated significantly enhanced proliferation on IA lysozyme nanofibrils at pH 5 (Supplementary Fig. 7). Excellent cell adhesion was observed on both the IA lysozyme monolayers and lysozyme nanofibrils due to the cationic surface inherited from the precursor lysozyme (Fig. 2a)^29^. In addition, IA lysozyme nanofibrils having a periodic β-sheet structure and hydrophobic surfaces, are capable of supporting cellular adhesion through lipid–fibril interactions^30^. Lysozyme does not present RGD sequences^31^. Cells are thought to secrete and deposit ECM proteins locally very soon after cultures are initiated^32^. Subsequent to the initially passive cell adhesion, cell spreading on liquid interfaces requires the binding of integrin to their nascent ECM proteins. This is demonstrated by the administration of soluble RGD (Arg-Gly-Asp) peptides as a competitive inhibitor to the binding of integrin with RGD-containing ECM (fibronectin and laminin)^32^. After the treatment with 2 mM soluble RGD peptides for 2 h, cell spreading was reduced across both groups of the IA lysozyme monolayers and lysozyme nanofibrils when compared with the non-treatment controls (Fig. 2b). Although significantly reduced, blocking of the integrin-fibronectin interaction did not completely suppress cell spreading, implying that binding interactions of integrin with other ECM proteins are involved in cell spreading at the liquid interface. Thus, an appropriate precursor lysozyme enables hMSC spreading at the liquid interface. Following initial passive adhesion, hMSCs interact with their nascent ECM proteins during adhesion and spreading at the liquid interface.

To assess the mechanotransduction pathways of hMSCs on the IA lysozyme monolayers and lysozyme nanofibrils, cell spreading was analyzed in the absence or presence of small molecule inhibitors of myosin-based contractility (blebbistatin), Rho-associated protein kinase (Y-27632) and actin polymerization (Cytochalasin D) (Supplementary Figs. 8, 9). Strikingly, all these inhibitors significantly reduced hMSC spreading (Fig. 2c, d). These results highlight that, cell spreading on the IA lysozyme monolayers and lysozyme nanofibrils is mediated through myosin-based contractility, Rho-associated kinase, and actin polymerization, consistent with our previously reported molecular clutch model^33^.

### Enhanced neuronal differentiation at IA lysozyme nanofibrils

**Fig. 3:**
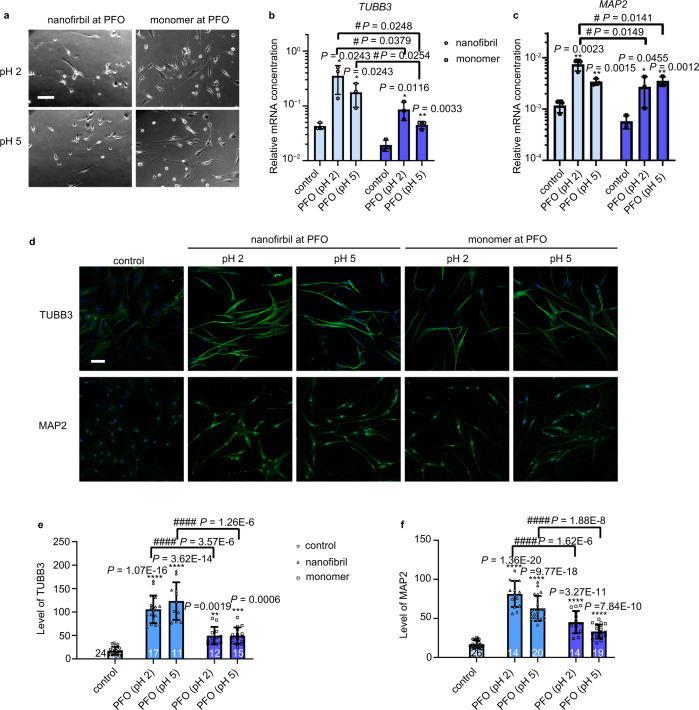
Enhanced neurogenesis of hMSCs on nanofibril networks at the PFO/water interface. **a** Phase contrast images of hMSCs after culturing at the PFO/water interface for 14 days. Scale bar: 100 μm. **b**, **c** Expression of neurogenesis markers *TUBB3* and *MAP2* after interfacial culture for 14 days. Cells cultured on coverslips as control. Gene expression was normalized to *GAPDH*. *n* = 3, mean ± s.d., *P < 0.05, **P < 0.01 versus control, ^#^*P* < 0.05, two-tailed Student’s *t*-test. **d** Immunocytochemistry confirms enhanced expression of neurogenesis markers at PFO compared with culturing on coverslips as control. Scale bar: 100 μm. **e**, **f** Quantification of the mean fluorescence intensity per cell for analysis of TUBB3 and MAP2 expression after interfacial culture for 14 d. Graphs show the number of cells from 2 biologically independent experiments; mean ± s.d., **P < 0.01, ***P < 0.001, ****P < 0.0001 versus control, ^####^*P* < 0.0001, one-way ANOVA with Bonferroni *post hoc* test.

Protein nanofibril was selected to clarify whether the fibrillary structure at adaptive liquid interface is responsible for the enhanced neuronal differentiation of hMSCs. hMSCs were cultured on IA lysozyme monolayer and lysozyme nanofibrils in growth medium for two weeks. Interestingly, hMSCs showed polarized neuron-like morphology at the liquid interface in the absence of any soluble neuronal differentiation inducing factors (Fig. 3a). The extent of neuronal differentiation was quantified by using quantitative reverse transcription polymerase chain reaction (qRT-PCR) for the neuronal markers β-tubulin III (*TUBB3*) and microtubule-associated protein 2 (*MAP2*). Relative to the lysozyme monomer coated coverslip as control, hMSCs on IA lysozyme monolayers exhibited significantly upregulated *TUBB3* and *MAP2* gene expression (Fig. 3b, c). Similarly, hMSCs on IA lysozyme nanofibrils exhibited significantly upregulated *TUBB3* and *MAP2* gene expression, compared with the lysozyme nanofibrils coated coverslip as control. Strikingly, hMSCs cultured on IA lysozyme nanofibrils at pH 2 showed the greatest differentiation. *TUBB3* and *MAP2* gene expressions of hMSCs cultured on IA lysozyme nanofibrils (pH 2) were significantly higher than those on the IA lysozyme monolayers. Immunocytochemistry further verified TUBB3 and MAP2 expression at protein level (Fig. 3d). TUBB3 and MAP2 protein expressions were significantly upregulated on IA lysozyme nanofibrils over those for the IA lysozyme monolayers (Fig. 3e, f). Also, the mature neuronal marker MAP2 was the most upregulated for hMSCs cultured on the IA lysozyme nanofibrils at pH 2, consistent with the gene expression finding. Taken together, these results indicate that protein nanofibrils with a fibrillary structure favor neuronal differentiation of hMSCs at liquid interface. The fibrillary structure at an adaptive liquid interface is responsible for the enhanced neuronal differentiation of hMSCs, in concordance with our previous result^16^.

### hMSCs adapt to liquid interface through lipid raft assembly

**Fig. 4:**
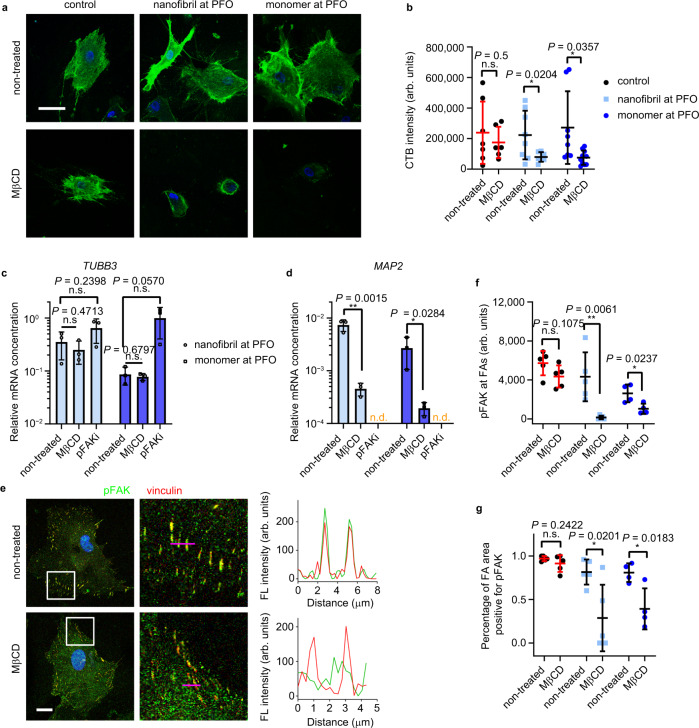
Cells rapidly adapt to fluid interface via lipid raft/FAK pathway, that direct neuronal differentiation of hMSCs. **a** Representative confocal images of lipid rafts of non-treated and MβCD-treated hMSCs. Cells cultured on coverslip as control. Lipid rafts were labeled with Alexa Fluor 488-conjugated CTB. Scale bar: 50 μm. **b** Quantification of CTB intensity. *P < 0.05, one-way ANOVA with Bonferroni *post hoc* test. Expression of neurogenesis markers *TUBB3* (**c**) and *MAP2* (**d**) of non-treated, MβCD-treated and pFAK inhibitor (pFAKi)-treated hMSCs cultured at the PFO/water interface (pH 2). Gene expression was normalized to *GAPDH*. *n* = 3, mea*n* ± s.d., *P < 0.05, **P < 0.01, two-tailed Student’s *t*-test. **e** Representative confocal images (magnifications in the second column) of the colocalization of pFAK and vinculin of non-treated and MβCD-treated hMSCs on protein nanosheets at the PFO/water interface (pH 2). Pink lines in the second column indicate the pixel regions for intensity profiles. Scale bars: 20 μm. **f** Quantification of pFAK localization to FA area by fluorescence intensity. **g** Quantification of percentage of FA area positive for pFAK. **f**, **g** *n* = 5 cells for control and nanofibril at PFO; *n* = 4 cells for monomer at PFO; Li*n*es and error bars indicate the mean ± s.d., *P < 0.05, **P < 0.01, one-way ANOVA with Bonferroni *post hoc* test.

Given that the early cell-matrix contact at the cell periphery can modulate the mechano-sensitive molecular pathways, we next sought to investigate the role of lipid raft in cell adhesion and spreading at a liquid interface. Lipid rafts, enriched in glycosphingolipids, cholesterol and membrane proteins, are tightly packed and ordered nanoscale domains floating in the cell membrane^34^. Lipid rafts serve as concentrating platforms that capture and reorganize the cytoskeletal machinery^35,36^. Therefore, they play a critical role in translating extracellular signals into downstream signaling and cell behaviors. We found that hMSCs at a liquid interface are very sensitive to treatment with methyl-β-cyclodextrin (MβCD), a cholesterol-sequestering agent that disrupts the integrity of lipid rafts^37^. Lipid rafts of hMSCs were specially stained with fluorescently–conjugated cholera toxin B subunit (CTB) (Fig. 4a). Noticeably, the intensity of CTB at the plasma membrane of hMSCs at the liquid interface was significantly decreased after treatment with a low dose of MβCD (25 μM), but not for hMSCs grown on coverslips as control (Fig. 4b). After three days culture with MβCD treatment, hMSCs were gradually formed agglomerated at the liquid interface, indicating no cell attachment. We postulate that the assemblies of lysozyme monomers and nanofibrils with cationic charge and amphiphilic nature have high affinities for the cell membrane and lipid-rich microdomains, which is important for cell adhesion and spreading at the liquid interface.

Considering the cell signaling role of lipid raft, we have further analyzed the connection between lipid raft, FAK phosphorylation and neuronal differentiation of hMSCs at a liquid interface by using chemical inhibitors. Remarkably, the disruption of lipid rafts by MβCD impairs the upregulation of neuronal differentiation markers *TUBB3* and *MAP2*, highlighting the connection between lipid raft integrity and neuronal differentiation of hMSCs (Fig. 4c, d). Lipid raft integrity is important to internalize cell adhesion molecules and recruit them to different plasma membranes^38,39^. Such polarized translocation has shown to direct axon growth and guidance^40^. On the other hand, in response to niche stimuli such as ECM proteins and mechanical signals, lipid rafts can coalesce into stable and large raft domains as concentrating platforms and form large signal transduction complexes. We found that the inhibition of lipid rafts with MβCD significantly reduces phosphorylated FAK (pFAK) expression at focal adhesion sites (Fig. 4e–g). The phosphorylation of FAK, an early downstream signal of integrin, can initiate a signal transduction cascade involving spatial activation of protein kinases and small GTPase proteins for the neuronal marker expression^41^. This was demonstrated by the observation that the FAK inhibitor 14, which blocks the phosphorylation of FAK, impairs the expression of mature neuronal marker *MAP2* (Fig. 4d). Moreover, as shown in Fig. 4f, pFAK expression is more significantly diminished by MβCD treatment on IA lysozyme nanofibrils than that on IA lysozyme monolayers. This indicates that phosphorylation of FAK probably largely relies on the integrity of lipid raft domains for hMSCs cultured on the IA lysozyme nanofibrils, whereas other FAK activation pathways might exist for that on IA lysozyme monolayers. Taken together, these data suggest that assembly of lysozyme monomers and nanofibrils at a fluid interface favors lipid raft formation and stability, triggering the phosphorylation of FAK, pathway activation, and neuronal differentiation.

To understand further the enhanced neuronal differentiation on IA lysozyme nanofibrils, we analyzed the expression of pFAK at focal adhesion sites. We found that hMSCs on the IA lysozyme nanofibrils (pH 2) displayed a lower level of FAK phosphorylation than those on IA lysozyme monolayers (Supplementary Fig. 10). Lower expression of pFAK leads to low microtubule stability, leading to a greater number of retraction events and, subsequently, axon extension as well as neuronal differentiation^42^. Therefore, hMSCs display increased neuronal differentiation on IA lysozyme nanofibrils (pH 2).

**Fig. 5:**
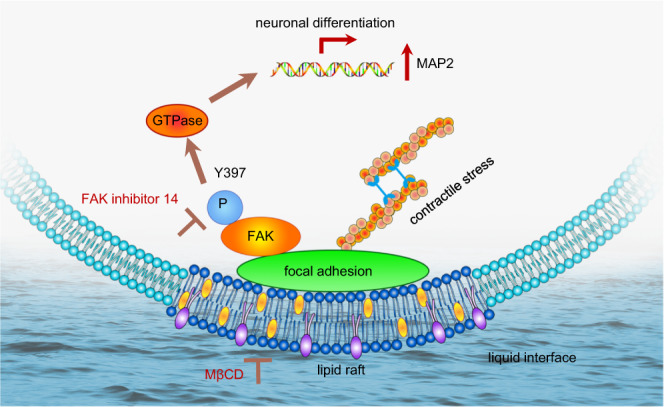
Schematic of adaptive liquid interface inducing neural differentiation of hMSCs via lipid raft assembly and FAK phosphorylation.

Our results show that protein assembly at a liquid interface provides a conceptually different method to prepare adaptive biomaterials. By coupling the fluidity of the liquid interface with dynamic features of protein assemblies, the IA lysozyme monolayer and lysozyme nanofibrils are sensitive to the cell traction forces. This provides a unique scenario to study the mechanism by which cells rapidly adapt to the continually dynamic microenvironment (Fig. 5). We found that lipid raft microdomains play a central regulatory role in both the initial cell adhesion and subsequent neuronal differentiation of hMSCs. First, lipid raft is involved in the process that internalizes cell adhesion molecules and recruits them to the different plasma membranes^43^. Second, lipid rafts function as concentrating platforms and prepare for the integration of large-signal transduction complexes^36^. These enable cells to adapt rapidly to the continually dynamic microenvironment. Furthermore, our results indicate that FAK is one of the important mechanosensors at adaptive liquid interfaces. Spatial and temporal regulation of FAK phosphorylation is required for neuronal differentiation of hMSCs. Overall, our study brings to light the previously unknown mechanism that links lipid raft formation and FAK phosphorylation to hMSC differentiation at an adaptive liquid interface.

We present an adaptive biomaterial based on two-dimensional protein nanoassemblies at a liquid–liquid interface. The adaptive features of protein assembly at a liquid interface can be easily adjusted by varying pH. Based on the cationic surface inherited from the precursor lysozyme, hMSCs can spread at the fluid interface according to the molecular clutch model without extra ECM protein coating. hMSCs cultured at IA lysozyme nanofibrils showed enhanced neuronal differentiation over protein monolayers at a liquid–liquid interface. Our results suggest that lipid raft plays a central role in directing neuronal differentiation of hMSCs at an adaptive fluid interface. hMSCs should adapt rapidly to dynamic microenvironments through lipid rafts in membrane microdomains with integration of downstream signals involving FAK which direct hMSC neurogenesis. Since native ECM exhibits dynamic adaptivity, this finding promises to unlock additional knowledge in the future to enhance our understanding of the basics of cell–ECM dynamic interactions and the underlying biophysics of mechanotransduction. In addition, the adaptive liquid interface adds a new dimension to the regulation of stem cell differentiation. By incorporating bioactive proteins or responsive polymers, the liquid interface might provide opportunities for the design of previously unimagined adaptive biomaterials for applications in regenerative medicine and tissue engineering.

## Methods

### Synthesis of protein nanofibril

A solution of lysozyme was prepared by dissolving lysozyme from hen egg white (80 mg, Sigma-Aldrich) in deionized water (4 mL). The solution pH was adjusted to 2.0 using aqueous HCl (2 M). After filtration using a 0.22 μm syringe-driven filter, the solution was heated at 90 °C for 20 h with stirring at 300 rpm. Large aggregates were removed by centrifugation at 2290 × *g* for 3 min. Nanofibril concentration was measured using Bio-rad protein assay kit with BSA as a standard.

### Fabrication of IA lysozyme monolayers and nanofibrils

In a typical experiment, PFO solvent (1 mL, Sigma-Aldrich) was added into individual chambers of a 24-well plate. The pH of the lysozyme solution (1 mg/mL) in 0.9% NaCl was adjusted to 2.0 or 5.0 using HCl (2 M). The solution was filtered (pore size, 0.22 μm) then added carefully on top of the PFO phase followed by incubation at 37 °C for 3 h. After incubation, the upper aqueous phase was replaced with PBS buffer by repeatedly rinsing. In this way, a lysozyme monolayer was formed at the liquid–liquid interface. Fibril nanosheets were fabricated using a similar procedure without the filtration step (Supplementary Fig. 11).

### Fluorescence labeling of proteins

Lysozyme (40 mg) was dissolved in pH 9.0 sodium carbonate buffer (100 mM, 20 mL). Fluorescein isothiocyanate (FITC, Sigma-Aldrich) dissolved in DMSO (1.0 mg/mL, 1 mL) was added dropwise to the lysozyme solution with stirring at 400 rpm. After incubation at 4 °C overnight, NH_4_Cl was added to a final concentration of 50 mM and was incubated for 2 h at 4 °C. The precipitate was collected by centrifugation at 9840 × *g* for 10 min and was then lyophilized for storage after washing with water.

### In situ fluorescence imaging analysis

FITC-lysozyme was used to form protein nanosheets at a liquid–liquid interface in a 48-well plate with observation using an upright fluorescence microscope (BX51, Olympus). Protein nanofibrils were labeled with Thioflavine T (ThT, 50 μM) and incubated at room temperature for 1 h. ThT-labeled protein nanofibrils were then used to form two-dimensional nanostructures at a liquid–liquid interface. The ThT-labeled protein nanofibrils were detected using an excitation wavelength of 440 nm.

### Atomic force microscope analysis

The protein or fibril nanosheets were transferred to silicon wafer according to our previously reported method^16^. Briefly, a silicon wafer was placed on the bottom of a 24-well plate chamber then PFO was added. After formation of protein or fibril nanosheets, the upper aqueous phase was rinsed sequentially with PBS buffer and deionized water. The resulting nanosheets were then transferred to a silicon wafer using a tweezer followed by air drying overnight. Topographic images of the dried protein or fibril nanosheets at a liquid–liquid interface were obtained using a Bruker Icon Dimension atomic force microscope using a standard silicon probe in tapping mode in air. Images were analyzed using the Gwyddion software. The coverage ratio of protein nanofibrils was calculated using the measurement function of area fraction in the Image J program (NIH).

### FTIR analysis

Lysozyme solution in 0.9% NaCl (2 mg/mL, 5 mL) was added to PFO in a glass vial. After vortex mixing for 10 s, the emulsion was allowed to stand at room temperature for 1 h. This led to formation of emulsion lower phase at the bottom of vial with an upper aqueous phase. The upper aqueous phase was exchanged 8 times with PBS buffer and then 5 times rinsed with deionized water. The emulsion was then lyophilized prior to FTIR characterization. FTIR spectra were collected using a Nicolet 670SX FT-IR spectrophotometer continuously purged with dry nitrogen gas and equipped with an attenuated total reflection (ATR) accessory (Smart iTX ATR diamond) and MCT detector.

### Interfacial tension analysis

Interfacial tension was measured using an optical contact angle meter (Drop master‐SA‐Cs1, Kyowa Interface Science Co., Ltd., Japan). A droplet of PFO (5 μL) was injected into a protein solution (1 mg/mL) while suspended from a dosing needle by using the pendant-drop method. The results were analyzed using the software of interface Measurement & Analysis System (FAMAS). The evolution of interfacial tension between PFO and water was recorded every 10 s following injection. Deformation of the PFO droplet was recorded using a digital camera.

### Culture of human mesenchymal stem cells

Human mesenchymal stem cells (Lonza, PT-2501), derived from bone marrow, were expanded in mesenchymal stem cell growth medium (Lonza, PT-3001) at 37 °C and 5% CO_2_. The growth medium includes both the basal media (PT-3238) and the growth supplement kit (PT-4105) for proliferation of human bone marrow-derived MSCs. The medium was exchanged for fresh medium every 3 d. Cells were expanded to passage 5 prior to use.

### Interfacial culture

The upper aqueous phase at the liquid–liquid interface was replaced with growth medium by repeated rinse and exchange. Cells were then seeded at the liquid–liquid interface with a density of 5000 cells per cm^2^. The medium was exchanged every 3 days by repeated exchange with fresh medium. For differentiation analysis, cells were cultured in growth medium for 2 weeks unless otherwise stated.

### Cell viability assay

The MTS assay (Promega) was used to assess cell viability. Briefly, hMSCs were cultured at a liquid-liquid interface in a 48-well plate for 1 d and 7 d. MTS reagent (30 μL) was added to the aqueous phase (300 μL), followed by incubation at 37 °C for 1 h. The aqueous phase (200 μL) was then carefully transferred to a clean chamber of a 96-well plate. Absorbance at 490 nm was recorded using a plate reader (Bio-Rad).

### Cell proliferation assay

For cell proliferation studies, EdU incorporation assay was performed following the manufacturer’s protocol (Click-iT Plus EdU Cell Proliferation Kit for Imaging, Life Technologies C10638). Briefly, hMSCs were cultured at a liquid-liquid interface in a 48-well plate. On day 2 of the culture, EdU solution was added to growth medium to a final concentration of 10 μM. On day 3 of the culture, cells were fixed using 4% paraformaldehyde in PBS for 15 min, and permeabilized using 0.5% Triton X-100 in PBS for 20 min. Cells were then incubated with 10 µM Alex Fluor 488-azide (Life Technologies) in 100 mM Tris buffer, 1 mM copper(II) chloride, and 100 mM ascorbic acid, pH 8.5 for 30 min at room temperature. Hoechst 33342 (1:2000) staining was applied at room temperature for 20 min, and images were collected using a fluorescence microscope (BX51, Olympus).

### Inhibitor study

For cell spreading studies, the following concentrations were used: 50 µM blebbistatin, 30 µM Y-27632, 1 µM Cytochalasin D (all from Wako, Japan) and 2 mM GRGDS (Peptide institute, Japan). Each of these reagents was added 2 h prior to analysis. For lipid raft and pFAK studies, 25 µM methyl-β-cyclodextrin (MβCD, Santa Cruz Biotechnology) was added when seeding the cells. MβCD was incubated for 2 days prior to analysis. For differentiation studies, 25 µM MβCD and 4 µM FAK inhibitor 14 (Sigma-Aldrich) were used. MβCD was incubated until day 14 and was exchanged every 3 d. FAK inhibitor 14 was incubated until day 2 and then was exchanged for fresh growth medium.

### Total RNA extraction and qRT-PCR

Total RNA was extracted according to our previously reported method^16^. A 24-well plate was used for the analysis of neuronal differentiation. The hMSCs were cultured at the interface between 1 mL of PFO and 1 mL of growth medium. After incubation for 2 weeks, growth medium was aspirated as much as possible from the upper aqueous phase, then 500 µL of PFO was removed by pipette from the bottom of the plate. Subsequently, TRIzol reagent (500 µL, Invitrogen Life Technologies) was added into each well. The lysate with PFO was pipetted up and down several times to homogenize. The whole lysate with PFO was collected in a new tube. The mixture was centrifuged at 12,000 × *g* for 15 min at 4 °C. The upper aqueous/TRIzol phase was collected and transferred into a new tube. Chloroform (0.2 mL) was added for lysis and followed by incubation at room temperature for 10 min. The sample was centrifuged at 12,000 × *g* for 15 min at 4 °C. The clear supernatant was collected and transferred into a new tube. Ethachinmate kit (Nippon Gene, 312-01791) was used to co-precipitate with the RNA. The obtained RNA pellet was air dried for 10 min, then residual genomic DNA was eliminated by DNase treatment (TaKaRa). Agencourt RNAClean XP kit (Beckman Coulter, A63987) was used to selectively bind RNA to paramagnetic beads to remove excess oligonucleotides, salts, and enzymes. The final clean RNA sample was quantified with Nanodrop with a A260/A280 ratio of ~2. The complementary DNA strand was synthesized on total RNA (300 ng) through reverse transcription reaction (PrimeScript RT reagent Kit, TaKaRa). Quantitative real-time polymerase chain reaction was performed on the LightCycler 480 system II (Roche). All reactions were carried out in a final volume of 15 μL using LightCycler 480 SYBR Green I Master kit (Roche) and assayed in triplicate. Primers used are listed in Supplementary Table 2. Quantitative mRNA expression level was analyzed using GAPDH as house-keeping gene by the comparative C_T_ method (2^−ΔCT^ method).

### Immunofluorescence staining

For confocal microscopy imaging, cells were transferred to a glass plate and immunostained using standard procedures as described previously^16^. Briefly, the cell sheet was fixed with 4% paraformaldehyde for 15 min and permeabilized using 0.2% Triton-X in PBS for 5 min. The sample was blocked in 5% BSA in PBS for 1 h at room temperature, incubated with primary antibodies for anti-beta III Tubulin rabbit polyclonal antibody (1:200; Abcam ab18207) and anti-MAP2 chicken polyclonal antibody (1:200, Abcam ab5392) at room temperature for 3 h, anti-vinculin mouse monoclonal antibody (1:200, Santa Cruz sc-73614) and anti-FAK pY397 rabbit polyclonal antibody (1:200, Life Technologies 44-624 G) at 4 °C overnight. Secondary antibodies were incubated at room temperature for 2 h with Alex Fluor goat anti-rabbit 488 (1:100, Life Technologies A11034), Alex Fluor chicken anti-mouse 488 (1:100, Life Technologies A21200), Alex Fluor goat anti-chicken 488 (1:100, Life Technologies A-11039) or Alex Fluor goat anti-rabbit 568 (1:100, Life Technologies A11010). For F-actin staining, Alexa Fluor 568 conjugated phalloidin (1:200, Life Technologies A12380) was incubated at room temperature for 1 h. After Hoechst 33342 (1:3000, Life Technologies 2584w) staining at room temperature for 20 min, glass slides were mounted using Slowfade antifade kit (Life Technologies 1597356). For image analysis of lipid rafts, cells were directly incubated with Cholera Toxin Subunit B conjugated with Alexa Fluor 488 (1:100, invitrogen C34775) at 4 °C for 20 min after twice rinsing with PBS. Cells were then fixed with 4% paraformaldehyde for 15 min at room temperature. Images were acquired using a Leica TCS-SP5 confocal laser scanning microscope with a 20× air objective or 63× oil objective. Images were analyzed by using the Image J software program (NIH) or LAS AF Lite (Leica) software.

### Statistical analysis and reproducibility

Statistical comparisons were performed using one-way analysis of variance (ANOVA) with Bonferroni’s* post hoc* testing or Student’s *t*-test where appropriate. Values are mean ± the standard deviation while error bars are standard deviations from independent experiments. *P* values of less than 0.05 were considered statistically significant. All statistical analyses were performed using Origin 9.1 software.

For experiments where representative micrographs are shown, experiments were repeated twice (Figs. 1c, e, 2a, 3a, d, 4a, e and Supplementary Figs. 2, 5, 7a, 8, 9, 10a) or three times (Fig. 1b, d, and Supplementary Fig. 1) with two biological repeats each time.

### Reporting summary

Further information on research design is available in the Nature Research Reporting Summary linked to this article.

## Supplementary information

Supplementary Information

Reporting Summary

## Data Availability

Source data are provided with this paper. The datasets generated and analyzed are reported in this paper and also available from the corresponding author upon appropriate request. Source data are provided with this paper.

## Source data

Source Data
