# Supplementary material for: Adaptive liquid interfaces induce neuronal differentiation of mesenchymal stem cells through lipid raft assembly
Source: Nat Commun. 2022 Jun 3;13:3110. doi: 10.1038/s41467-022-30622-y (PMC9166733; doi:10.1038/s41467-022-30622-y)
Supplement: Supplementary file 1 — Supplementary Information [file 41467_2022_30622_MOESM1_ESM.pdf]

Supplementary Materials for

Adaptive liquid interfaces induce neuronal differentiation of mesenchymal stem cells through lipid raft assembly

Xiaofang Jia, [1*] Jingwen Song,[2,3] Wenyan Lv,[1] Jonathan P. Hill,[2] Jun Nakanishi,[4*] and Katsuhiko Ariga[2,3*]

*Corresponding author. Email: jiaxf5@mail.sysu.edu.cn (X. J.), NAKANISHI.Jun@nims.go.jp (J. N.), ARIGA.Katsuhiko@nims.go.jp (K. A.)

This PDF file includes:

Supplementary Figs. 1 to 11

Supplementary Table 1 to 2

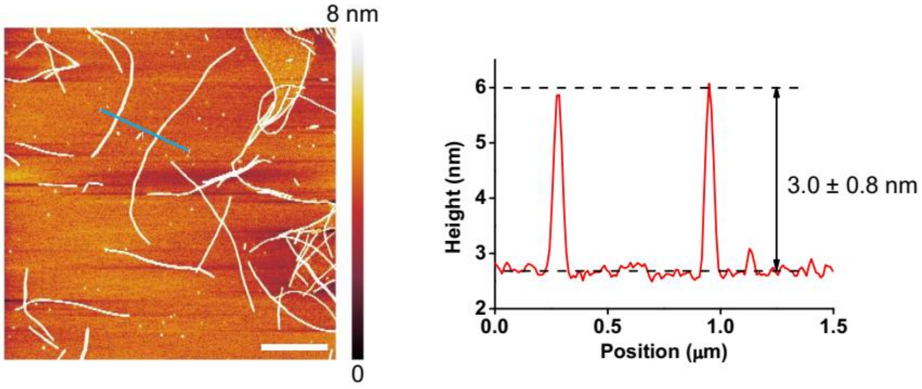

Supplementary Fig. 1 DFM images and the corresponding height profiles of protein nanofibrils. Scale bar: 1 μm.

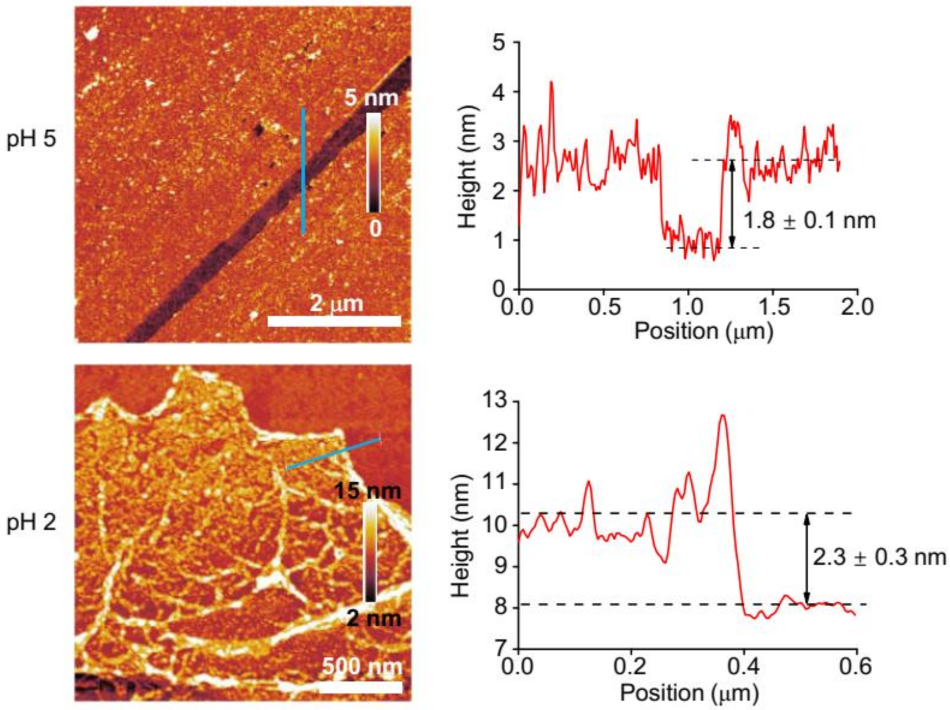

Supplementary Fig. 2 DFM images and the corresponding height profiles of protein nanosheets assembled at the PFO/water interface at pH 5 and pH 2.

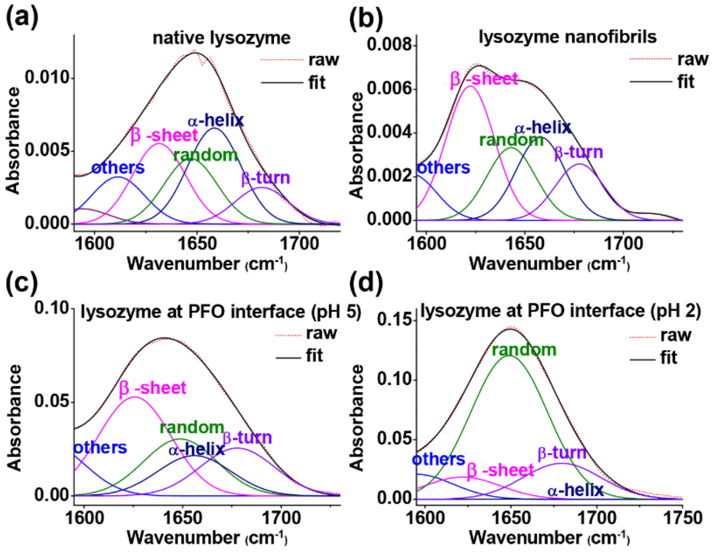

Supplementary Fig. 3 ATR-FTIR spectra of (a) native lysozyme solution, (b) lysozyme nanofibrils solution, and (c, d) dried lysozyme nanosheets assembled at the PFO interface at (c) pH 5 and (d) pH 2. Curve fitting of amide I spectra was carried out using Peakfit software.

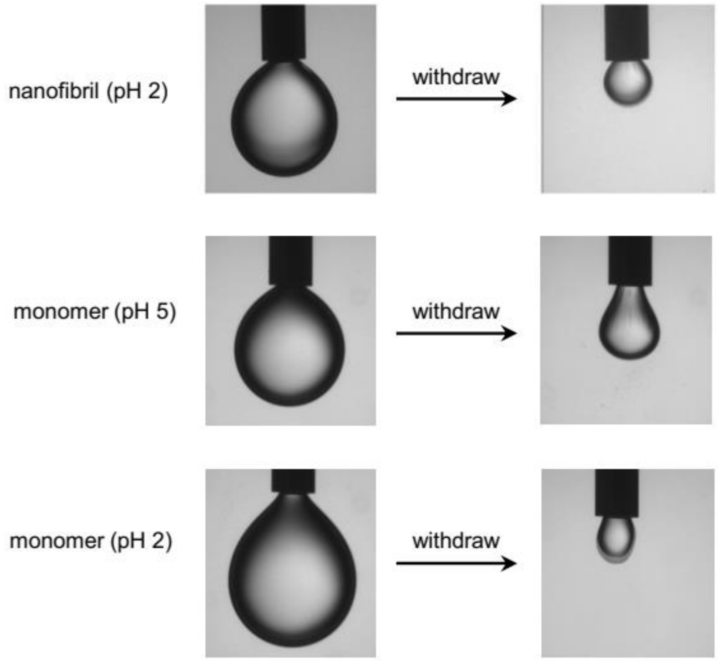

Supplementary Fig. 4 Images of the snapshots during PFO phase is withdrawn from the droplet in the bath of lysozyme nanofibrils solution at pH 2, lysozyme monomer solution at pH 5 or lysozyme monomer solution at pH 5.

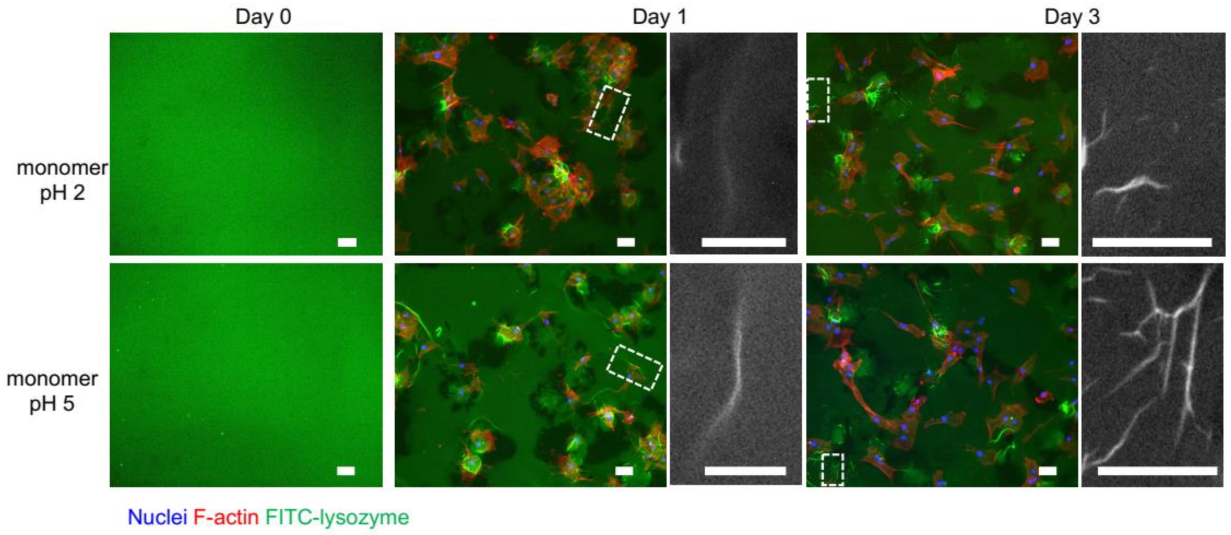

Supplementary Fig. 5 Protein nanosheets at liquid interface adapt dynamically to cell traction forces. Time-series images of protein nanosheets assembled at different pH before cell seeding (Day 0) and following remodeling by hMSCs after 1 d and 3 d. Dashed rectangle designates region shown with FITC-lysozyme channel isolated on the right. Nuclei in blue, FITC-lysozyme in green, F-actin in red, scale bar: 40 μm.

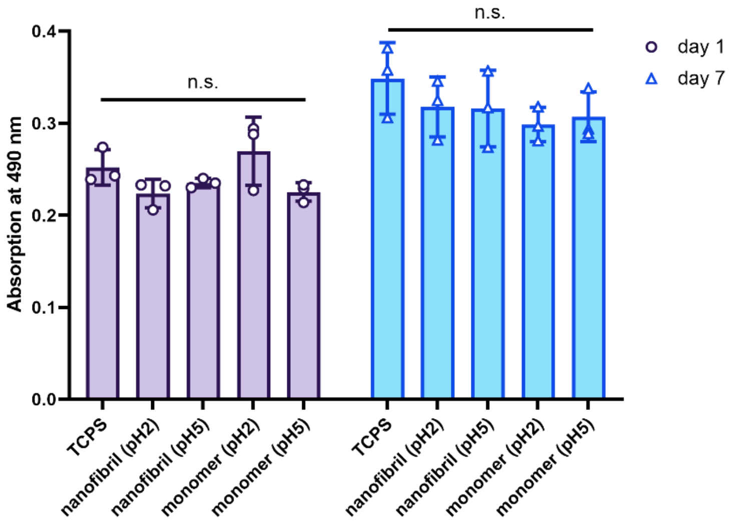

Supplementary Fig. 6 Cell viability of hMSCs after 1 day and 7 days culture on the monolayer of lysozyme nanofibril and monomer at the PFO interface using MTS assay. hMSCs cultured on tissue culture polystyrene (TCPS) as control. $n = 3$, mean ± s.d., two-tailed Student's t-test.

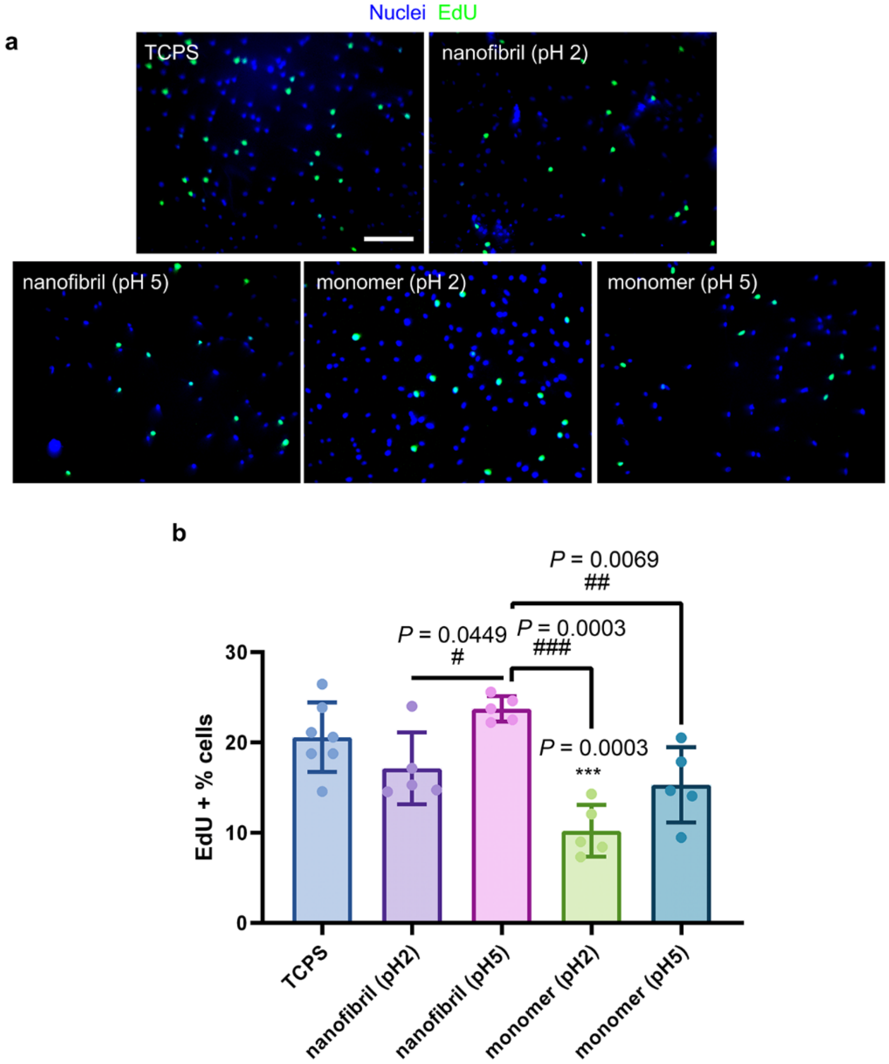

Supplementary Fig. 7 Cell proliferation between day 2 and day 3 culture on the monolayer of lysozyme nanofibril and monomer at PFO interface. a Representative immunofluorescence images of hMSCs after EdU incorporation for 24 h. Nuclei in blue, EdU in green, scale bar :100 μm. hMSCs cultured on tissue culture polystyrene (TCPS) as control. **b** Percentage of hMSCs positive for EdU staining. $^{***}P < 0.001$ *vs* TCPS, $^{\#}P < 0.05$, $^{\#\#}P < 0.01$, $^{\#\#\#}P < 0.001$, one-way ANOVA with Turkey's test, each data point represents an independent slide from two independent experiments.

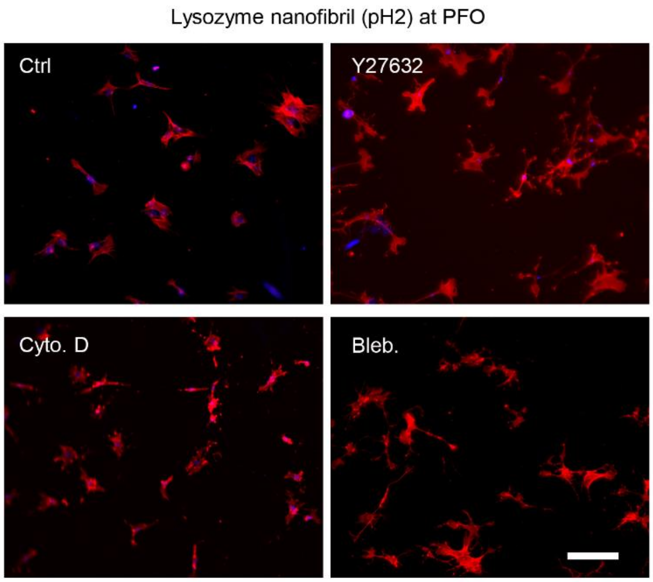

Supplementary Fig. 8 Representative fluorescence images of hMSCs on the monolayers of lysozyme nanofibrils at the PFO/water interface at pH 2 after 2 h of no treatment (control), or treatment with an inhibitor.

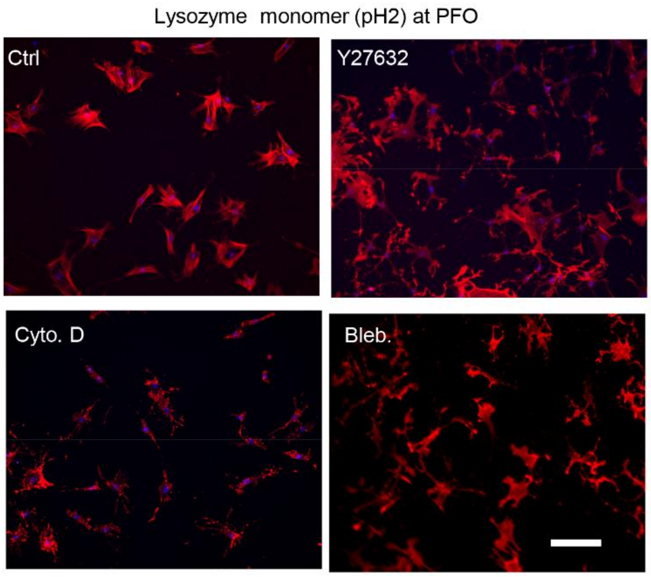

Supplementary Fig. 9 Representative fluorescence images of hMSCs on the monolayers of lysozyme monomers at the PFO/water interface at pH 2 after 2 h of no treatment (control), or treatment with an inhibitor.

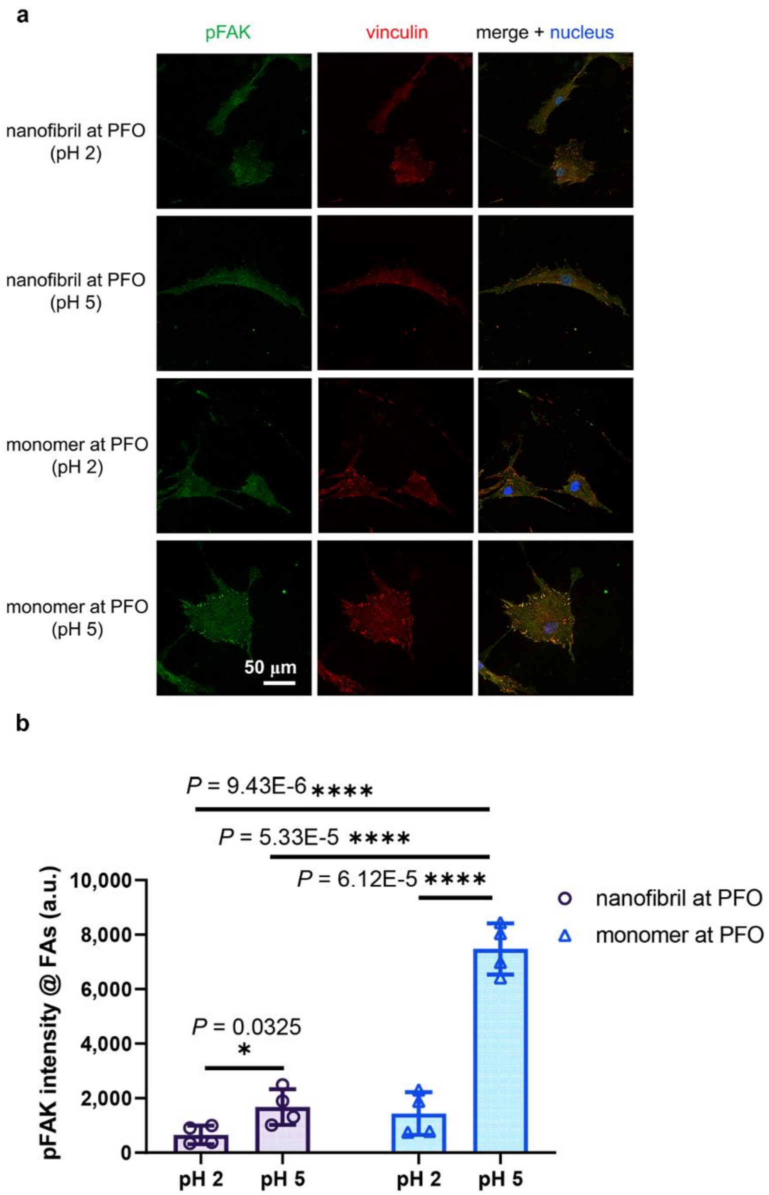

Supplementary Fig. 10 The expression of pFAK at focal adhesion sites of hMSCs at PFO interface. **a** Representative immunofluorescence confocal images of hMSCs at the PFO interface. green: pFAK, red: vinculin, blue: nuclei, scale bar: 50 μm. **b** pFAK intensity at focal adhesion sites of hMSCs at the PFO interface. a.u.: arbitrary units. Bars and error bars indicate the mean ± s.d. $n = 4$, $*P < 0.05$, $****P < 0.0001$, one-way ANOVA with Bonferroni post hoc.

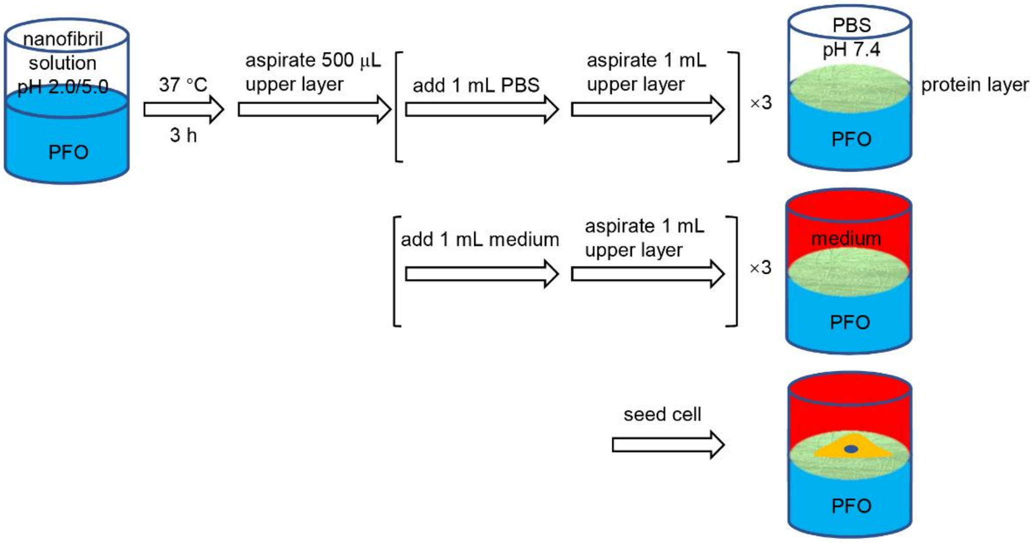

Supplementary Fig. 11 Schematic of cell culture on two-dimensional networks of protein nanofibrils at a liquid–liquid interface.

Supplementary Table 1. Percentage distributions of lysozyme secondary structures and the area ratio of amide I to amide II band before and after interaction with PFO as measured by ATR-FTIR spectroscopy.

protein	secondary structure					area ratio (amide I/II)
	α-helix	β-sheet	β-turn	random	others	
native lysozyme in solution	28.2	23.6	10.8	19.2	18.3	1.20
lysozyme nanofibrils	22.8	36.8	15.5	19.9	5.0	1.39
lysozyme at PFO (pH 5)	15.4	37.2	18.4	21.6	7.6	1.25
lysozyme at PFO (pH 2)	0.2	10.3	17.0	66.9	5.7	1.81

Supplementary Table 2. Primer sequences for RT-qPCR used in this work.

Gene	Forward primer	Reverse primer
GAPDH	TCA ACG GAT TTG GTC GTA TTG GG	TGA TTT TGG AGG GAT CTC GC
TUBB3	AGC CAG CAG TGT CTA AAC CC	GGG AGG ACG AGG CCA TAA AT
MAP2	CCA CCA GGT CAG AGC CAA TT	CTT CTT CTC ACT CGG CAC CA
