# Supplementary material for: Adaptive liquid interfaces induce neuronal differentiation of mesenchymal stem cells through lipid raft assembly
Source: Nat Commun. 2022 Jun 3;13:3110. doi: 10.1038/s41467-022-30622-y (PMC9166733; doi:10.1038/s41467-022-30622-y)
Supplement: Supplementary file 2 — Reporting Summary [file 41467_2022_30622_MOESM2_ESM.pdf]

nature portfolio

Corresponding author(s): Xiaofang Jia, Jun Nakanishi, Katsuhiko Ariga

Last updated by author(s): Apr 25, 2022

Reporting Summary

Nature Portfolio wishes to improve the reproducibility of the work that we publish. This form provides structure for consistency and transparency in reporting. For further information on Nature Portfolio policies, see our Editorial Policies and the Editorial Policy Checklist.

Statistics

For all statistical analyses, confirm that the following items are present in the figure legend, table legend, main text, or Methods section.

n/a	Confirmed	
☐	☒	The exact sample size (*n*) for each experimental group/condition, given as a discrete number and unit of measurement
☐	☒	A statement on whether measurements were taken from distinct samples or whether the same sample was measured repeatedly
☐	☒	The statistical test(s) used AND whether they are one- or two-sided<br>*Only common tests should be described solely by name; describe more complex techniques in the Methods section.*
☒	☐	A description of all covariates tested
☐	☒	A description of any assumptions or corrections, such as tests of normality and adjustment for multiple comparisons
☐	☒	A full description of the statistical parameters including central tendency (e.g. means) or other basic estimates (e.g. regression coefficient) AND variation (e.g. standard deviation) or associated estimates of uncertainty (e.g. confidence intervals)
☐	☒	For null hypothesis testing, the test statistic (e.g. *F*, *t*, *r*) with confidence intervals, effect sizes, degrees of freedom and *P* value noted<br>*Give P values as exact values whenever suitable.*
☒	☐	For Bayesian analysis, information on the choice of priors and Markov chain Monte Carlo settings
☒	☐	For hierarchical and complex designs, identification of the appropriate level for tests and full reporting of outcomes
☒	☐	Estimates of effect sizes (e.g. Cohen's *d*, Pearson's *r*), indicating how they were calculated

Our web collection on statistics for biologists contains articles on many of the points above.

Software and code

Policy information about availability of computer code

Data collection	LightCycler 480 II (Roche) was used to acquire and analyze quantitative real-time RT-PCR data (LightCycler 480 software version 1.5).<br>Leica TCS-SP5 confocal laser scanning microscope was used to acquire confocal images (LAS AF Lite (Leica) software or ImageJ, NIH).<br>Olympus BX51 fluorescence microscope was used to acquire in situ fluorescence images and phase contrast images at liquid interface (ImageJ, NIH).<br>Plate reader (Bio-Rad) was used to assess cell viability.<br>Bruker Icon Dimension atomic force microscope was used to acquire atomic force microscope images (Gwyddion software).<br>Nicolet 670SX FT-IR spectrophotometer was used to acquire ATR FTIR spectra. Curve fitting of amide I spectra was carried out using Peakfit software.<br>Optical contact angle meter (Drop master-SA-Cs1, Kyowa Interface Science Co., Ltd., Japan) was used to acquire interfacial tension data. The results were analyzed with the software of interface Measurement & Analysis System (FAMAS).
Data analysis	Origin 9.1 and Graphpad Prism 9 software for Windows was used for statistical analysis.<br>LightCycler 480 software version 1.5 was used to acquire and analyze quantitative real-time RT-PCR data.<br>LAS AF Lite (Leica) 2.6 software was used to acquire and analyze confocal images.<br>Image J software version 1.52a was used for quantifications of immunoflurescence images.<br>Gwyddion software version 2.60 was used to analyze dynamic force microscope images.<br>Peakfit software version v4.12 was used to analyze FTIR data.<br>Interface Measurement & Analysis System (FAMAS) software version 7.1.0 was used to analyze interfacial tension data.

For manuscripts utilizing custom algorithms or software that are central to the research but not yet described in published literature, software must be made available to editors and reviewers. We strongly encourage code deposition in a community repository (e.g. GitHub). See the Nature Portfolio guidelines for submitting code & software for further information.

March 2021

Data

Policy information about availability of data

All manuscripts must include a data availability statement. This statement should provide the following information, where applicable:

- Accession codes, unique identifiers, or web links for publicly available datasets
- A description of any restrictions on data availability
- For clinical datasets or third party data, please ensure that the statement adheres to our policy

Source data are provided with this paper. The datasets generated and analyzed are reported in this paper and also available from the corresponding author upon appropriate request.

Field-specific reporting

Please select the one below that is the best fit for your research. If you are not sure, read the appropriate sections before making your selection.

☒ Life sciences ☐ Behavioural & social sciences ☐ Ecological, evolutionary & environmental sciences

For a reference copy of the document with all sections, see nature.com/documents/nr-reporting-summary-flat.pdf

Life sciences study design

All studies must disclose on these points even when the disclosure is negative.

Sample size	No statistical methods were used to predetermine sample sizes. Sample size were chosen based on our previous experience to reliably measure experimental parameter and previous publications (i.e. Dupont et al. Nature 2011). The sample size chosen were sufficient to determine significance in all the assays, with reproducible statistically significant difference between conditions in all the experiments.
Data exclusions	No data was excluded.
Replication	The number of biological replications is indicated in Figure captions. The number of technical replications is at least two. All attempts at replication were successful.
Randomization	Randomization of animals is not relevant to this study.
Blinding	Cell culture experiments were not blinded. But data quantification was performed in a blinded fashion using batch names in order to avoid bias. All other analysis and data acquisition techniques were analyzed non-blinded because of practical reasons and the availability of robust and reproducible measurement and data analysis tools.

Reporting for specific materials, systems and methods

We require information from authors about some types of materials, experimental systems and methods used in many studies. Here, indicate whether each material, system or method listed is relevant to your study. If you are not sure if a list item applies to your research, read the appropriate section before selecting a response.

Materials & experimental systems

n/a	Involved in the study	
☐	☒	Antibodies
☐	☒	Eukaryotic cell lines
☒	☐	Palaeontology and archaeology
☒	☐	Animals and other organisms
☒	☐	Human research participants
☒	☐	Clinical data
☒	☐	Dual use research of concern

Methods

n/a	Involved in the study	
☒	☐	ChIP-seq
☒	☐	Flow cytometry
☒	☐	MRI-based neuroimaging

Antibodies

Antibodies used	beta III Tubulin mAb (Abcam, ab1820); MAP2 mAb (Abcam, ab5392); vinculin mAb (Santa Cruz, sc-73614); FAK pY397 mAb (Life Technologies 44-624G); Cholera Toxin Subunit B (Invitrogen, C34775);Alex Fluor goat anti-rabbit 488 (Life Technologies, A11034), Alex Fluor chicken anti-mouse 488 (Life Technologies, A21200), Alex Fluor goat anti-chicken 488 (Life Technologies, A-11039) or Alex Fluor goat anti-rabbit 568 (Life Technologies, A11010)
Validation	Each primary antibody was validated by manufacturer. Extensive antibody-validation data for each species and application, with relevant citations, are provided in each vendor's product website.

March 2021

Eukaryotic cell lines

Policy information about cell lines

Cell line source(s)	human derived mesenchymal stem cells procured from Lonza
Authentication	cells were authenticated by manufacturer and maintained phenotype over the duration (multiplication number) of our studies (ability to differentiate).
Mycoplasma contamination	Cells were tested and tested negative for mycoplasma contamination.
Commonly misidentified lines (See ICLAC register)	no commonly misidentified cell lines used.
